# NMR Spectroscopy of Macrophages Loaded with Native, Oxidized or Enzymatically Degraded Lipoproteins

**DOI:** 10.1371/journal.pone.0056360

**Published:** 2013-02-15

**Authors:** Paul Ramm Sander, Markus Peer, Margot Grandl, Ulrich Bogdahn, Gerd Schmitz, Hans Robert Kalbitzer

**Affiliations:** 1 Institute of Biophysics and Physical Biochemistry, University of Regensburg, Regensburg, Germany; 2 Centre of Magnetic Resonance in Chemistry and Biomedicine (CMRCB), Regensburg, Germany; 3 Department of Neurology, University of Regensburg, Regensburg, Germany; 4 Institute for Clinical Chemistry and Laboratory Medicine, University of Regensburg, Regensburg, Germany; Spanish National Cancer Center, Spain

## Abstract

Oxidized and enzymatically modified low-density lipoproteins (oxLDL and eLDL) play a key role in early stages of atherogenesis. Their uptake by recruited macrophages leads to endolysosomal phospholipidosis or foam cell formation, respectively, each of which is preceded by highly differential lipid restructuring processes. We applied ^1^H-NMR spectroscopy (NMRS) to elucidate these structural rearrangements both in consequence of lipoprotein modifications and following phagocytosis. Being specifically sensitive to the mobile lipid subset, NMRS of oxLDL and eLDL revealed a partial and total immobilization of lipids, respectively. NMRS of intact macrophages showed a sixfold increase in mobile lipids in case of loading with eLDL but no significant changes for oxLDL or native LDL. This finding reflected the disparate lipid storage in lipid droplets and in multilamellar endolysosomal clusters when loaded with either eLDL or oxLDL, respectively. Moreover, a significant shift of the degree of saturation towards mainly polyunsaturated fatty acid chains was found for the mobile lipid pool in eLDL-loaded macrophages. Additional analyses of lipid extracts by NMRS and mass spectrometry (MS) reflected these changes in lipid content and in fatty acid composition only partially. In summary, in-cell NMRS represents a unique lipidomics tool to investigate structural changes within the mobile lipid pool following atherogenic triggers that can be not detected by the analysis of lipid extracts by MS or NMRS.

## Introduction

Atherosclerosis is one of the most frequent causes of death around the world. Constituting the starting point of the majority of cardiovascular diseases, atheromatous plaques in arterial walls are the main reasons for hypertension, angina pectoris, myocardial infarction and stroke because of both a decrease of the functional inner diameter of vessels and an additional risk of thrombosis. It is an accepted fact that during the early stage of the development of atherosclerosis, low-density lipoproteins (LDL) enter the arterial intima where they become modified, i.e. oxidized or enzymatically degraded. In contrast to native LDL, these modified LDL are readily incorporated by macrophages via distinct mechanisms [1], [2]. In case of oxidized LDL (oxLDL), the cellular uptake gives rise to endolysosomal phospholipidosis, whereas the uptake of enzymatically-modified LDL (eLDL) ultimately leads to so-called foam cells, i.e. macrophages almost completely filled with neutral lipid droplets [3]. In the end, both events result in atheromatous plaques composed of entrapped macrophages, inflammatory cells, lipid droplets and cholesterol crystals.

Both the modification of LDL and the uptake by macrophages are accompanied by structural changes of these lipid assemblies. Native LDL is composed of about 3000 lipid molecules and one single copy of apoB-100, one of the biggest monomeric proteins in nature (ca. 550 kDa). At physiological temperatures, LDL is assumed to exhibit a core-shell structure [4] wherein the core is composed of neutral lipids (mainly cholesterylesters, but also free cholesterol and some triacylglycerols) in a liquid oil-like state, and the shell is comprised of amphiphilic phospholipids (mainly phosphatidylcholine and sphingomyelin) interleaved by free cholesterol molecules [5]. Upon mild oxidation, LDL particles undergo partial stripping of the polar surface layer, aggregate and rarely fuse [6], whereas enzymatic degradation gives rise to multilamellar liposomes consisting of membrane layers arranged in an onion-like fashion with oily inclusions [7]. In contrast, within macrophages, oxLDL is restructured to multilamellar aggregates stored in endolysosomes, whereas multilamellar eLDL are rapidly degraded and converted to cytosolic quasi-fluid lipid droplets [3].

NMR spectroscopy (NMRS), the only non-invasive methodology implying high-throughput potential at atomic resolution, has already been utilized to study lipoproteins for decades [8], [9], [10]. Due to its sensitivity to chemical and structural differences, NMRS facilitates the accurate quantification of different lipoprotein classes and subclasses directly from unprocessed plasma/serum [8], [9], [10]. Moreover, NMRS metabolomics/metabonomics approaches do not only yield absolute lipoprotein quantities but also succeeded in analyzing metabolite concentrations and even molecular lipid compositions [11], [12]. Mass spectrometry (MS) based lipidomics, on the other hand, is widely accepted for high sensitivity qualitative and quantitative lipid species analysis of extracted lipids [13].

In addition to the quantitative analysis of extracted lipids, NMRS of unprocessed samples provides a unique insight into the mobility of molecules and thus into the structure of molecular assemblies like lipoproteins [14]. The mobility of a molecule is reflected by its correlation time which directly affects the relaxation rate of NMRS resonances and thus also their line widths. Rigid assemblies of molecules result in very broad resonances whose intensities are below the noise level and thus are not resolved by most common NMRS applications. For instance, resonances of lipids constituting cellular membranes are mostly NMR-invisible due to their relative rigidity [15]. Even *in vivo*, localized NMRS of mammalian brain does not show significant lipid signals [16], [17] unless certain diseases, e.g. brain tumors, cause lipid droplets via necrosis, apoptosis or other forms of cellular stress [18], [19], [20], reviewed in [21]. These stress-induced NMR-visible lipid droplets have also been observed in a huge variety of cell types *in vitro*, including several tumor cell lines [22], [23], [24], [25], neural progenitor cells [26], [27], cancer stem cells [28], and macrophages [29].

Concerning the structural transition of LDL particles upon modification, NMRS results on oxLDL have been published agreeing that spectra of oxLDL show decreased and line broadened lipid signals compared to native LDL [30], [31]. Enzymatic modifications of LDL were reported to result in fusion of lipid particles when treated with α-chymotrypsin [6], and showed a shift from phosphatidylcholine to lyso-phosphatidylcholine accompanied by a decrease in *bis*-allylic methylene in fatty acid chains in case of addition of phospholipase A_2_ [32].

In the following, we will focus on the lipid metabolism of macrophages visible by NMR in living cells. Using macrophages as model system we have studied the NMR-visible structural and compositional changes that occur after uptake of lipoproteins by macrophages and discuss them in the context of data obtained by other biophysical methods.

## Materials and Methods

### Isolation of Low Density Lipoprotein (LDL)

Lipoproteins were isolated from fresh, nonlipemic human plasma of healthy donors by a modified sequential preparative ultracentrifugation in KBr gradients (d = 51.025 g/ml to 1.052 g/ml) followed by extensive dialysis and filter sterilization according to published methods [33], [34]. All lipoprotein concentrations mentioned are protein concentrations determined by Lowry method. Lipoprotein fractions were stored in the presence of 0.5 mmol/l EDTA at 4°C. None of the regular blood donors had diabetes mellitus or treatment for arterial hypertension.

### Modification of LDL

eLDL was generated under sterile conditions. LDL was diluted to 2 mg/ml protein in PBS (w/o Ca2+, Mg2+). To 5 ml LDL diluted in PBS (2 mg/ml), 6.6 µg/ml trypsin (Sigma, Germany) and 400 µg/ml cholesterylester hydrolase (Seikagaku, Japan) were added. Subsequently the solution was incubated at 37°C for 48 h.

Mildly oxidized LDL was achieved by dialyzing purified LDL fractions (1 mg protein/ml) against 5 µM CuSO4 for 40 h. The oxidation process was stopped by dialysis in PBS/EDTA. Further extensive dialysis in PBS was made. Afterwards, the oxLDL was sterile filtered and protein content was determined by Lowry method. The mild oxidation of LDL was controlled by electrophoresis.

### Macrophage Cell Culture and Lipoprotein Treatment

Macrophage isolation and cell culture were conducted as previously described [35]. Briefly, human macrophages were derived from monocytes, which had been isolated by leukapheresis followed by counterflow elutriation. None of the regular blood donors had diabetes mellitus or treatment for arterial hypertension. Phagocytic differentiation of monocytes to macrophages was conducted by culturing monocytes in macrophage serum-free medium (Invitrogen, Germany) at 10^6^ cells/ml in tissue culture plates (6-well flat bottom; Sarstaedt, Germany) in a growth chamber (5% CO_2_, 37°C) and with monocyte-colony stimulating factor (M-CSF, 50 ng/ml, R&D Systems, USA). After four days, macrophages were loaded with LDL-species for 48 hours followed by harvesting. In addition to native low-density lipoproteins (LDL, 40 µg/ml), enzymatically modified LDL (eLDL, 40 µg/ml) and mildly oxidized LDL (oxLDL, 80 µg/ml) were used for LDL-loading of macrophages.

### Mass Spectrometry

Lipid extraction and mass spectrometry based targeted lipid analysis in the presence of isotopically labeled or not naturally occurring lipid species as internal standards was performed essentially as described previously [36], [37], [38], [39] for at least six replicants of each LDL subtype (N = 6 for macrophage extractions, N = 11 for extractions of pure lipoprotein samples). Direct flow injection utilized a 1200 series binary pump (Agilent, Waldbronn, Germany) coupled via electrospray ionization (ESI) to a Quattro Ultima tandem mass spectrometer (Micromass, Manchester, UK). Reversed phase and HILIC LC-ESI-MS/MS was conducted using a 1200 series binary pump and a hybrid triple quadrupole linear ion trap mass spectrometer API 4000 Q-Trap (Applied Biosystems, Darmstadt, Germany). Quantification was performed by standard addition calibration to cell homogenates or plasma using a number of naturally occurring lipid species for each lipid class. To calculate total amounts of saturated, mono- and poly-unsaturated lipid species, the following lipid classes were considered (see Table S1): phosphatidylcholine (PC), -ethanolamine (PE), -serine (PS), -inositol (PI), lyso-phosphatidylcholine (LPC), sphingomyeline (SM), ceramide (Cer) and cholesterylester (CE).

### NMR Spectroscopy

For NMR spectroscopy of lipoproteins, the apoB-100 concentration of native LDL was used as a reference for scaling to the lipid content of the LDL solutions. The samples (N = 3 for LDL and oxLDL, N = 2 for eLDL) contained 0.9–3.7 mg protein equivalent per ml, 10% deuterium oxide (D_2_O) and 40 µM dimethyl-silapentane-sulfonate (DSS, internal standard, 0.00 ppm). 500 µl of the samples were transferred to standard 5 mm NMR tubes (502, Norell Inc., USA). During measurement, the temperature was kept at 25°C. Measurements were performed at high resolution NMR Bruker Avance 600 and 800 MHz spectrometers employing a one-dimensional NOESY pulse sequence (Bruker nomenclature “noesygppr1d”) with low power presaturation at the water resonance frequency during the relaxation delay of 5 s and during the mixing time of 10 ms. At least 32 scans with 64 K datapoints and 7.7 s repetition time were accumulated. After exponential line broadening by 0.3 Hz, the raw data were Fourier-transformed (64K datapoints), followed by a manual phase and baseline correction.

For NMR spectroscopy of macrophage suspensions, 3 million cells per sample (N ≥4) were washed twice in phosphate-buffered saline (PBS), resuspended in 500 µl PBS solution containing 10% D_2_O and 40 µM DSS and transferred to a 5 mm Shigemi NMR tube (500 µl, Shigemi, USA) that allows sedimentation within the sensitive volume of the probe coil due to a susceptibility-matched solid glass bottom. Samples were cooled to 5°C and NMR measurement started within less than 15 min thereafter. During measurement, the temperature was kept at 5°C in order to prevent rapid degradation processes of the cells due to e.g. absence of nutrition, accumulation of secreted/leaked molecules, and hypoxia. NMR spectra were acquired employing a gradient-based water suppression pulse sequence (Bruker nomenclature “zgesgp”) with additional adiabatic presaturation [40]. Sixty-four scans with 32 K datapoints and 3.7–4.7 s repetition time were accumulated followed by an exponential line broadening of 2 Hz. After Fourier transformation (32 K data points), the spectra were phase- and baseline-corrected manually.

For NMR spectroscopy of total cellular lipids, neutral cellular lipids were extracted from macrophages by means of a chloroform-methanol protocol (N = 3, except for eLDL-loaded macrophages, N = 2). Briefly, 3 million cells were washed twice in 500 µl phosphate-buffered saline (PBS) and subsequently dried overnight in a centrifugal evaporator (RC1010, Juoan Inc., USA). The precipitate was dissolved in 700 µl chloroform-d1/methanol-d4 (2∶1 vol/vol) supported by 20 min sonication on ice. After centrifugation for 10 min at 4°C and 16 060 g, the clear supernatant was transferred to a standard 5 mm NMR tube (502, Norell Inc., USA). In order to account for extractions of lipophilic components of the sample cup material, blank sample cups without cells were also included in the extraction procedure. NMR spectra were acquired using a basic pulse-acquire pulse sequence (Bruker nomenclature “zg”). Eight scans with 32 K datapoints and 14.6 s repetition time were accumulated at 5°C. After exponential windowing (1 Hz) and subsequent Fourier transformation (32K data points), a manual phase and baseline correction was performed.

### Quantitative Evaluation of NMR Spectra

In case of NMRS of macrophage suspensions, NMR-visible lipids were quantified by deconvolution as described previously [26], [28]. In case of cell suspensions, the deconvolution approach outperforms simple integration of defined spectral regions, since in NMR spectra of cell suspensions, the resonances of interest, i.e. the lipid peaks, are heavily overlapped by signals of other intra- and extracellular molecules, e.g. NMR-visible proteins and lactate, respectively. Briefly, the spectral region between 0.5 ppm and 3.1 ppm was fit using a standard Matlab (The MathWorks, Natick, MA) fit routine by means of mixed Gaussian-Lorentzian multiplets at published chemical shifts (Table S2) of small metabolites (e.g. amino acids and lactate) [41], [42] and NMR-visible macromolecules, i.e. mobile lipids [19], [21], [22], [24] and mobile proteins [43], [44], [45]. A representative outcome of this deconvolution procedure is presented in Fig. S1. Prior to deconvolution, all spectra were normalized to the integral over the protein NH-region between 7 ppm and 9 ppm and/or over the glutathione signal at 2.55 ppm, which was similar to normalization to the protein NH-region but resulted in more reliable quantities for spectra acquired with a presaturation water suppression pulse sequence due to a partial saturation transfer from water to exchangeable protein NH-protons during presaturation. Thus, this normalization procedure reflects the amount of intracellular proteins, which is believed to be a more reliable scaling to cell number compared to a simple cell counting prior to NMR sample preparation since both the process of funneling the cell suspension into the NMR tube and the heterogeneous sedimentation of cells into the sensitive coil volume of the NMR spectrometer probe within the Shigemi tube during acquisition are likely to distort the actually acquired cell quantity. The total amount of NMR-visible lipids is expressed as the integral over the deconvolved mobile lipid peak “-CH_3_” dominated by contributions of fatty acid terminal methyl protons and thus a quantity reflecting the amount of NMR-visible fatty acid chains (esterified and unesterified). The degree of polyunsaturation is expressed as the ratio of the integral over the *bis-*allylic methylene protons of fatty acid chains (i.e. “-CH = CH-CH_2_-CH = CH-” at 2.8 ppm) divided by the integral over “-CH_3_” and subsequently multiplied by 3/2 to account for the different numbers of protons contributing to “-CH = CH-CH_2_-CH = CH-” and “-CH_3_” per methylene and methyl group, i.e. two and three, respectively. Thus, this quantity reflects the average number of *bis-*allylic methylene per fatty acid chain, and can be interpreted as a marker for the degree of polyunsaturation.

In case of NMRS of extracted lipids, a sophisticated normalization was not required since the extraction started with 3 million cells in all cases. Spectra were scaled to the integral over the methyl signal of the internal standard (trimethylsilane, TMS) at 0.0 ppm. The spectra were corrected for extracted sample cup components, e.g. plasticizers, by subtracting the spectra of the blank samples (*vide supra*) from the spectra of the cell extracts. The total amount of extracted lipids was quantified by means of the integral over the spectral region dominated by protons of the terminal methyl group of fatty acid chains, i.e. between 0.8 ppm and 0.95 ppm. The relative amount of *bis-*allylic methylene per fatty acid is derived from the integral over the spectral region between 2.75 ppm and 2.9 ppm, which is divided by the integral over the terminal methyl region.

Statistically significant differences between control macrophages and loaded macrophages were determined using Mann-Whitney *U* test.

## Results

### NMRS of Low-density Lipoproteins

**Figure 1:**
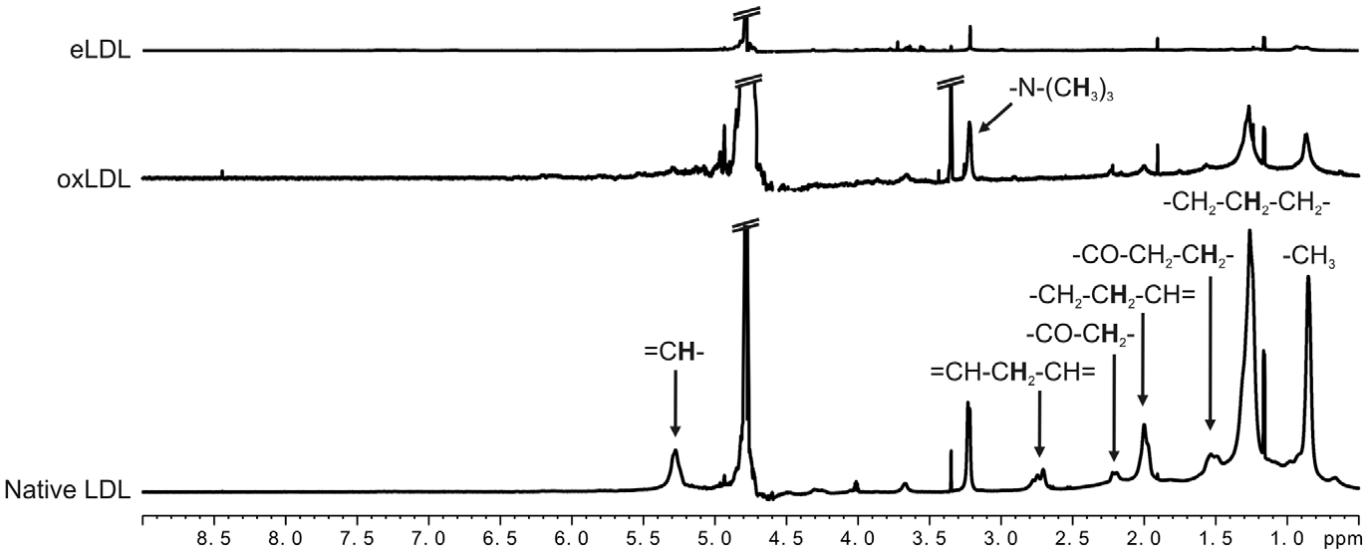
Representative NMR spectra of native LDL, oxLDL and eLDL. (Bottom) Native LDL gave rise to lipid signals predominantly of methyl and methylene protons of fatty acid chains. (Center) NMR spectra of oxLDL showed substantially decreased lipid signals compared to native LDL. (Top) eLDL spectrum: resonances of lipids were virtually absent. Instead, amino acid signals and mobile protein peaks were observed. NMR spectral parameters: 800 MHz spectrometer frequency, 1D-NOESY pulse sequence with water presaturation (noesygppr1d), 7.7 s repetition time, 10 ms mixing time, temperature: 25°C. Spectral annotation according to dominant contributions to NMR signals. Spectra were normalized to the concentration of apoB-100 determined prior to oxidation or modification. Each LDL subtype was investigated on the basis of at least three replicates from different donors, N = 3 except for “oxLDL” (N = 2).

NMR spectroscopy of native LDL, oxidized LDL (oxLDL) and enzymatically modified LDL (eLDL) yielded completely different spectral features (Fig. 1) that were consistent within the replicates of each LDL subtype. In case of native LDL, characteristic signals of methyl and methylene protons of fatty acid chains were found. Regarding oxLDL, oxidation led to a significant decrease in lipid signal intensities compared to native LDL. Concurrently, a line broadening was evident for lipid resonances but not for other signals, e.g. peaks of impurities such as isopropanol at 1.17 ppm and acetic acid at 1.91 ppm. In contrast, NMR spectra of eLDL samples were virtually devoid of any lipid signals. Instead, signs of protein degradation could be identified, i.e. signals of amino acids (e.g. multiplets of methyl protons of valine, leucine and isoleucine between 0.9 ppm and 1.1 ppm) and resonances of mobile protein residues at typical random coil chemical shifts (e.g. M1 at 0.9 ppm, M2 at 1.2 ppm, M3 at 1.4 ppm etc., following the nomenclature of Kauppinen *et al.* [43] and Behar *et al.* [44], [45]). It is important to note that all NMR spectra were normalized to the concentration of the apolipoprotein apoB-100 that was determined before oxidation with copper sulfate or treatment with trypsin and cholesterylesterase. Therefore, Fig. 1 reflects the NMR-visibility of fatty acid chains at equal total fatty acid concentrations.

### NMR-visible Lipids in Macrophages

**Figure 2:**
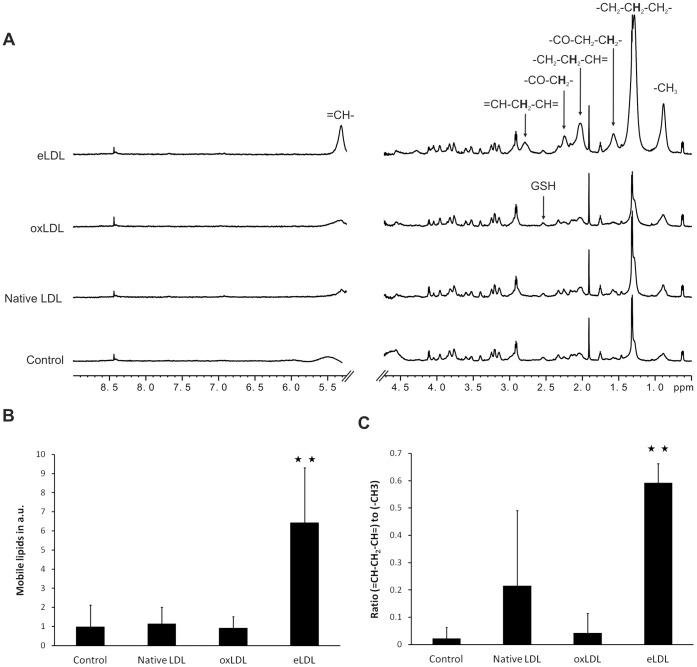
Changes in NMR-visible lipids as consequence of loading macrophages with modified LDL. (A) Moderate mobile lipid signals were present in NMR spectra of native macrophages (control). Loading with native LDL or oxLDL did not result in any significant increase of mobile lipid intensities, whereas eLDL loading gave rise to a dominant lipid signal increase. NMR spectral parameters: 800 MHz spectrometer frequency, gradient-based water suppression pulse sequence (zgesgp) with additional water presaturation, 3.7 s repetition time, temperature: 5°C. Spectra were normalized to protein and/or glutathione (GSH) signal intensities. Spectral annotation according to dominant contributions to NMR signals. (B) The NMR-visible lipid content, i.e. the integral in arbitrary units over the deconvolved NMR signal of the terminal methyl group of fatty acid chains. (C) The average percentage of *bis-*allylic methylene per fatty acid chain in control macrophages and macrophages loaded with native LDL, oxLDL and eLDL, i.e. the ratio of NMR-visible *bis-*allylic methylene groups and methyl groups. Deconvolved peaks of (-CH = CH-CH_2_-CH = CH-)- and (-CH_3_)-protons were integrated. Mean and standard deviation of five samples from different donors, N = 5 except for “LDL” (N = 4). Mann-Whitney *U* test: **p<0.01.

NMR spectra of macrophage suspensions exhibited typical features characterizing NMR spectroscopy of whole cells (Fig. 2), i.e. sharp resonances of small molecules, e.g. of amino acids, lactic acid and acetic acid, and broad signals of NMR-visible macromolecules that can be classified into mobile lipid and mobile protein resonances. Despite a slight increase in mobile lipid intensities, the appearance of NMR spectra of macrophages loaded with native LDL did not change significantly compared to that of unloaded macrophages. Likewise, loading with oxidized LDL only gave rise to minor spectral changes compared to control macrophages. Interestingly, similarly to the findings obtained for pure oxLDL (*vide supra*), the lineshapes of the lipid resonances were broader than in all other NMR spectra of macrophages. However, in contrast to loading with native LDL or oxLDL, eLDL had profound effects on macrophage spectra. First, the overall content of NMR-visible fatty acid chains was increased six- to sevenfold (Fig. 2B). Second, the relative amount of different methylene moieties within NMR-visible fatty acids changed substantially. Most strikingly, per fatty acid chain, the number of the *bis-*allylic methylene groups, which are specific for polyunsaturated fatty acids, increased upon loading with eLDL from 0.02 to 0.59 groups on average (Fig. 2C).

### Lipophilic Extraction of Macrophages

**Figure 3:**
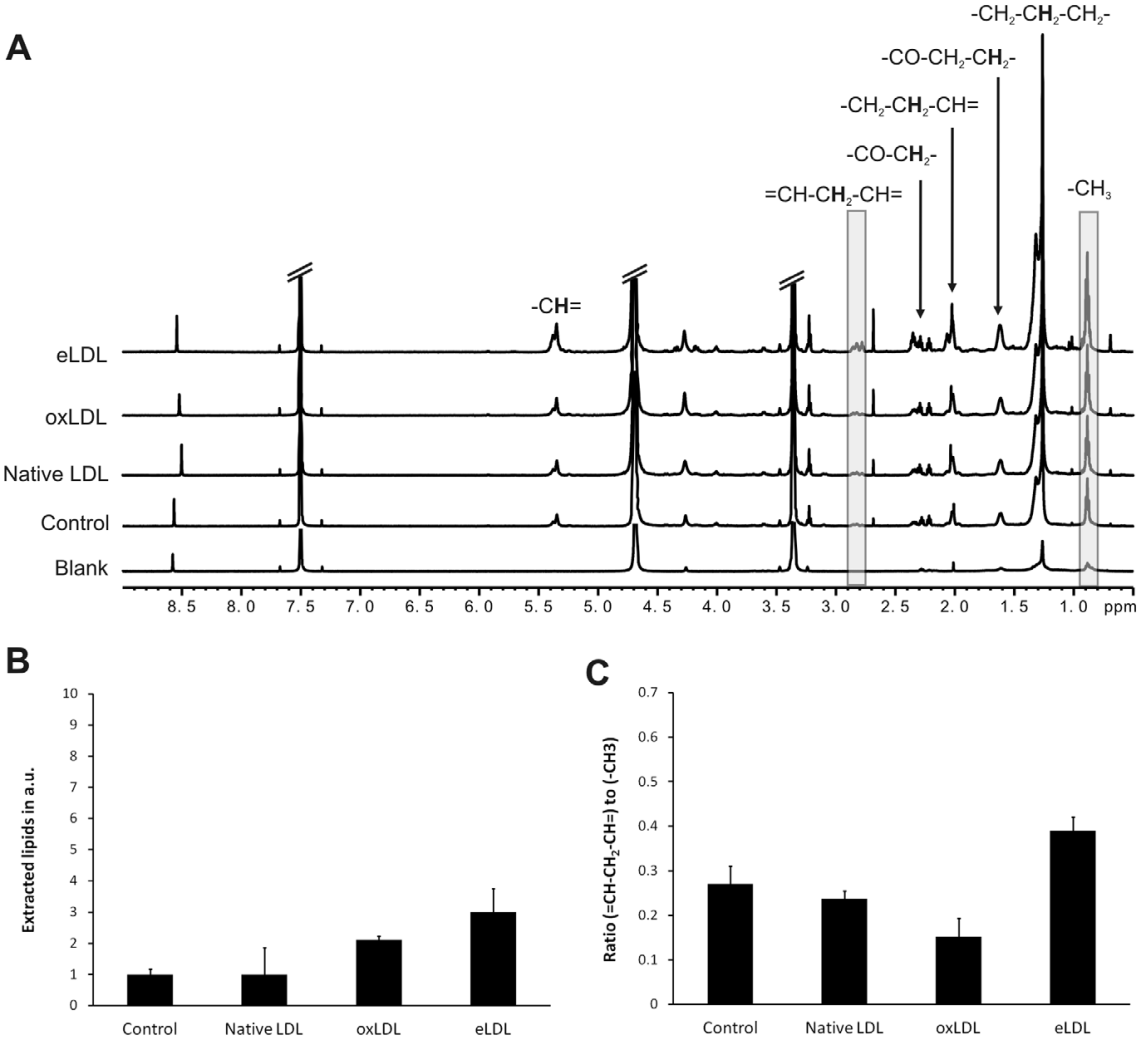
Effects of modified LDL on lipid extractions of macrophages. (A) NMR spectra of extracted lipids. “Blank”: Extracted components of the sample cup (This spectrum is subtracted from cell extract spectra prior to quantification). Shaded regions were used for quantification. NMR spectral parameters: 800 MHz spectrometer frequency, pulse-acquire pulse sequence (zg), 14.6 s repetition time, temperature: 5°C. Spectral annotation according to dominant contributions to NMR signals. (B) The total intracellular lipid content, i.e. the in arbitrary units over the spectral region between 0.8 ppm and 0.95 ppm dominated by protons of the terminal methyl group of fatty acid chains. (C) The average percentage of *bis-*allylic methylene per fatty acid chain in control macrophages and macrophages loaded with native LDL, oxLDL and eLDL, i.e. the ratio of *bis-*allylic methylene groups and methyl groups. Spectral regions of (-CH = CH-CH_2_-CH = CH-)- and (-CH_3_)-protons were integrated (2.75–2.9 ppm and 0.8–0.95 ppm, respectively). Mean and standard deviation of three samples from different donors except for “LDL” (N = 2).

In order to compare NMR-visible lipids in intact macrophages with the total amount of intracellular lipids, macrophage suspensions were extracted by means of a lipophilic chloroform-methanol extraction procedure which has been shown to bring in solution both lipophilic and amphiphilic lipid species, e.g. cholesterylester and phospholipids, respectively. NMR spectroscopy of these extracted lipids revealed a trend of increase in the concentration of fatty acid chains only in case of macrophages loaded with eLDL (Fig. 3). However, the increase was only threefold and not sixfold as for NMR-visible intracellular lipids (*vide supra*). Evaluation of the degree of saturation of the fatty acid chains also unveiled a small trend of increase in unsaturation in case of eLDL loading. Again, this increase was rather small (from 0.27 to 0.39 *bis-*allylic methylene groups per fatty acid chain and from 0.79 to 0.95 double bonds per fatty acid chain, the latter calculated from the integral over the “CH = CH” peaks around 5.4 ppm; data not shown) compared to the severalfold increase observed by NMRS of macrophage suspensions (from 0.02 to 0.59 *bis-*allylic methylene groups per fatty acid chain*;* the 5.4 ppm region could not be evaluated reliably). Loading with native LDL or oxLDL did not result in significant changes in extractable lipids, neither regarding the total amount nor the degree of unsaturation.

**Figure 4:**
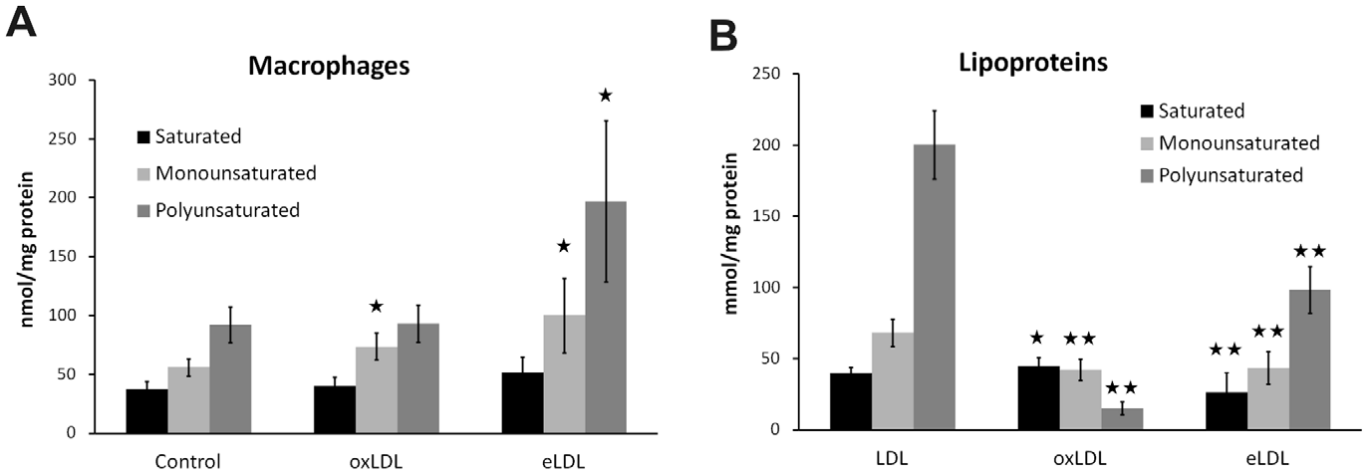
ESI-MS/MS analysis of lipoprotein loaded macrophages and native LDL, oxLDL and eLDL. (A) eLDL induced significant accumulation of mono- and polyunsaturated lipid species in macrophages. oxLDL only elevated levels of monounsaturated species. (B) Lipid compositional analysis of lipoproteins. oxLDL showed substantially decreased levels of mono- and polyunsaturated lipids compared to native LDL. Saturated lipid species were decreased in eLDL, but increased in oxLDL. Mean and standard deviation of six (A) or eleven (B) replicates from different donors. Mann-Whitney *U* test: *p<0.05, **p<0.01.

Qualitative and quantitative lipid analysis was also performed using targeted ESI mass spectrometry. Phospholipids, sphingolipids and cholesterylester species were analyzed in lipoprotein loaded macrophages after lipid extraction (Fig. 4A) and in lipoproteins (Fig. 4B). Whereas saturated and monounsaturated species were almost equal in LDL, eLDL and oxLDL particles, polyunsaturated species were differentially distributed, being the major species in LDL and eLDL and only the minor species in oxLDL. eLDL loading led to a significant increase of both mono- and polyunsaturated lipid species in macrophages, whereas oxLDL led to a minor but significant increase only in the monounsaturated species.

## Discussion

We could show that oxidation and enzymatic modification of low-density lipoproteins changed the NMR-visibility of their fatty acid chains significantly. NMR spectra of oxLDL showed highly decreased fatty acid methylene and methyl signals, and in eLDL, fatty acids were virtually NMR-invisible. The uptake by macrophages also had very diverse effects on NMR-visible lipids. Whereas in case of loading with native LDL and oxLDL significant changes could not be observed neither in whole cell NMR spectra nor in spectra of extracted lipids, eLDL loading resulted in a massive increase of NMR-visible lipids accompanied by a shift towards polyunsaturated mobile fatty acid chains. Both the increase of lipid signals and the degree of polyunsaturation were reflected only partially in NMR spectra and mass spectra of lipid extracts.

Polyunsaturated alkyl chains are especially prone to oxidative processes. The decrease of lipid signals as consequence of oxidation of LDL has already been reported by other groups [30], [31]. In line with their argumentation, a general shift from a liquid-like state of the LDL core to a more rigid assembly of lipids due to e.g. cross-linking of fatty acid chains by oxidation products such as aldehydes, could explain the overall decrease in NMR-visibility. However, it cannot be excluded that part of the intensity reduction and especially of the line broadening could also be caused by residual paramagnetic copper binding to oxLDL. Concerning eLDL, to the authors’ best knowledge there has not been any report on NMR spectroscopy of LDL enzymatically modified by trypsin and cholersterylesterase yet. The finding that fatty acids within eLDL are virtually NMR-invisible is not surprising since the multilamellar structure of eLDL [46] nicely resembles plasma membranes that are also composed of phospholipid bilayers interleaved by cholesterol and that are also too rigid to be detected by conventional NMR spectroscopy, e.g. using localized spectroscopic techniques *in vivo* [16], [17] or investigating whole cell suspensions *in vitro* [15].

Since the overall concentration of fatty acid chains should not change during modification of LDL, the NMR-invisibility of the major part of lipids in oxLDL and eLDL can be attributed to changes of their structural arrangement and their internal mobility. This finding highlights the advantage of NMR spectroscopy over other methods that solely determine the total lipid content and are not able to resolve structural properties, e.g. mass spectrometry.

Structural transformations of modified lipoproteins during the uptake by macrophages have already been reported. Oxidized LDL is known to induce extensive endolysosomal phospholipidosis in macrophages, i.e. persistent storage of lipids within endolysosomes wherein an elevated pH impedes further digestion of oxLDL components [3], [47]. Interestingly, those entrapped and only partly degraded lipids are largely structured in a multilamellar fashion. The NMR-invisibility of these lipids could be explained by these multilamellar structures resembling rigid plasma membrane arrangements. In contrast, eLDL was shown to induce rapid and extensive foam cell formation [3], [47], i.e. large amounts of cytoplasmic lipid droplets are present in macrophages loaded with eLDL. In turn, lipid droplets, due to their liquid-like core consisting of neutral lipids, are known to be NMR-visible to a high degree [18], [19], [22], [23]. Therefore, the significant increase of NMR-visible lipids upon eLDL loading could be explained by the fact that eLDL lipids become mobile as a consequence of restructuring the multilamellar eLDL assembly into lipid droplets after phagocytosis and during reesterification processes.

Additionally, we found that eLDL-induced mobile lipids exhibited a significantly higher percentage of polyunsaturated fatty acid chains compared to those mobile lipids already present in control macrophages prior to loading. A shift to polyunsaturated mobile lipids has been reported to be correlated to apoptosis *in vivo* [48], [49]. However, the experimental conditions, i.e. apoptotic solid tumors *in vivo* on the one hand, and on the other hand eLDL-loaded macrophages *in vitro*, are too different to draw the conclusion that eLDL induces apoptosis in macrophages. Further investigations are required in order to elucidate the nature of a higher degree of polyunsaturation in the NMR-visible lipid pool upon eLDL loading.

Both the elevated lipid content and the shift towards polyunsaturated fatty acid chains occurred predominantly in the mobile lipid compartment as conducted from the fact that the changes in both NMRS and MS analyses of extracted lipids were less pronounced than for the NMR-visible lipid pool in intact cells. In contrast to other lipidomics methodologies, NMR spectroscopy is able to discriminate between mobile droplet-like and rigid membrane-like structures, and thus constitutes a unique tool to study changes that preferentially occur in the mobile lipid compartment of intact cells.

It is important to discuss that temperature also crucially affects the structure of LDL particles. Around 30°C, the LDL core exhibits a structural phase transition between a liquid oil-like state at higher temperatures and a highly ordered smectic-like liquid crystal state at lower temperatures [50]. Since rigid structures are virtually NMR-invisible, this phase transition is reflected in a gradual decrease of intensities of lipid signals with decrease of the temperature [14]. In addition, the transition temperature decreases with the relative concentration of unsaturated fatty acids that is the contribution of NMR-visible resonances increases at a given temperature. In our study, the cell suspensions had to be kept at 5°C during NMRS measurements in order to prevent rapid degradation. Therefore, it is likely that intracellular lipid assemblies were also partially NMR-invisible due to the low temperature in conjunction with a putative phase transition. However, we found a severalfold increase of intracellular lipid signals at 5°C which was induced by loading with eLDL whose lipid content was totally NMR-invisible even at higher temperature, i.e. 25°C. Thus, our line of argument for an NMR-detectable restructuring process of modified LDL upon uptake by macrophages remains coherent. Moreover, the increase of intracellular lipid signals in case of loading with eLDL might even be underestimated at low temperatures. Further investigations at physiologic temperature, enabled by a sophisticated perfusion setup that ensures optimum supply of oxygen and nutrients during the measurement within the NMR spectrometer, will shed light on the absolute effect size of LDL loading on the NMR-visiblity of intracellular lipids.

In conclusion, structural changes of low-density lipoproteins as consequence of atherogenic modifications were readily detectable by NMR spectroscopy. Additionally, the differential uptake of these modified LDL species by macrophages followed by specific digestion and restructuring processes could also be monitored. Endolysosomal phospholipidosis induced by oxidized LDL had no significant effects on NMR spectra of intact cells, whereas eLDL-induced cytosolic lipid droplet formation could readily be detected in terms of immense growth of mobile lipid signals. The NMR-visibility of lipid resonances, i.e. the mobility of the corresponding fatty acid chain moieties, reflected the structural modifications of oxLDL and eLDL as well as their differential storage in macrophages to a much higher degree than conventional NMR-spectroscopic and mass-spectrometric analyses of total cellular lipids, i.e. via extraction, regarding both lipid content and the degree of saturated, monounsaturated and polyunsaturated lipid species. This finding underlines the benefit of NMR spectroscopy as a lipodomics tool to specifically monitor the mobile lipid pool that is clearly different from the total lipid pool. For the interpretation of in-cell and in-vivo NMRS data it is important to realize that invisibility of mobile lipids not necessarily means the absence of a significant intracellular lipid pool.

## Supporting Information

Figure S1 **Deconvolution of NMR spectra of macrophage suspensions.** Top: Measured spectrum (thin line) and result of a lorentzian-gaussian fit (thick line). Second from top: Fit components of small molecules, e.g. lactate (lac), alanine (ala), dimethyl-silapentane-sulfonate (DSS), acetate (ace), glutathione (GSH). Third from top: Fit components of NMR-visible lipids, i.e. mobile lipids (ML). Bottom: Model spectrum of NMR-visible proteins, i.e. mobile proteins (MP).
(TIF)

Table S1 Individual lipid species for analysis of saturation level by mass spectrometry. Lipid species abbreviations: Phosphatidylcholine (PM), phosphatidylethanolamine (PE), phosphatidylserine (PS), phosphatidylinositol (PI), lysophosphatidylcholine (LPC), sphingomyeline (SM), ceramide (Cer), cholesterylester (CE).
(DOCX)

Table S2 Resonance assignment used for deconvolution of NMR spectra of macrophage suspensions. Lineshape abbreviations: singlet (s), doublet (d), multiplet (m), broad peak treated as singlet (b), multiplet treated as broad singlet (m/b).
(DOCX)
