# Supplementary figures and images for: NMR Spectroscopy of Macrophages Loaded with Native, Oxidized or Enzymatically Degraded Lipoproteins
Source: PLoS One. 2013 Feb 15;8(2):e56360. doi: 10.1371/journal.pone.0056360 (PMC3574142; doi:10.1371/journal.pone.0056360)

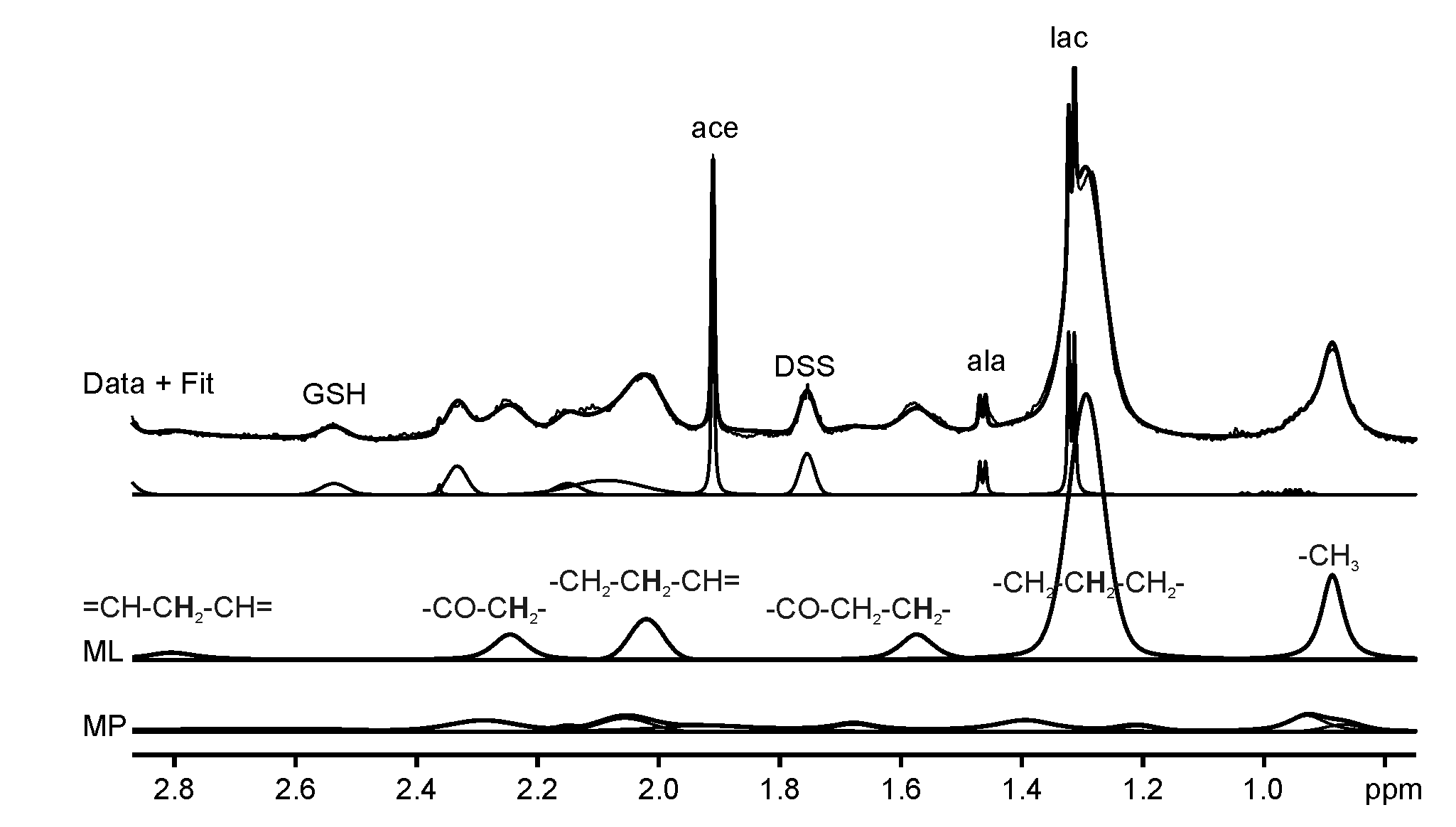

Supplement: Figure S1 — Deconvolution of NMR spectra of macrophage suspensions. Top: Measured spectrum (thin line) and result of a lorentzian-gaussian fit (thick line). Second from top: Fit components of small molecules, e.g. lactate (lac), alanine (ala), dimethyl-silapentane-sulfonate (DSS), acetate (ace), glutathione (GSH). Third from top: Fit components of NMR-visible lipids, i.e. mobile lipids (ML). Bottom: Model spectrum of NMR-visible proteins, i.e. mobile proteins (MP). (TIF) [file pone.0056360.s001.tif]
