# Supplementary material for: NMR Spectroscopy of Macrophages Loaded with Native, Oxidized or Enzymatically Degraded Lipoproteins
Source: PLoS One. 2013 Feb 15;8(2):e56360. doi: 10.1371/journal.pone.0056360 (PMC3574142; doi:10.1371/journal.pone.0056360)
Supplement: Table S1 — Individual lipid species for analysis of saturation level by mass spectrometry. Lipid species abbreviations: Phosphatidylcholine (PM), phosphatidylethanolamine (PE), phosphatidylserine (PS), phosphatidylinositol (PI), lysophosphatidylcholine (LPC), sphingomyeline (SM), ceramide (Cer), cholesterylester (CE). (DOCX) [file pone.0056360.s002.docx]

## Supporting Table S1

**Individual lipid species for analysis of saturation level by mass spectrometry.** Lipid species abbreviations: Phosphatidylcholine (PM), phosphatidylethanolamine (PE), phosphatidylserine (PS), phosphatidylinositol (PI), lysophosphatidylcholine (LPC), sphingomyeline (SM), ceramide (Cer), cholesterylester (CE).

| **Lipid class** | **PC** | **PE** | **PS** | **PI** | **LPC** | **SM** | **Cer** | **CE** |
| --- | --- | --- | --- | --- | --- | --- | --- | --- |
| Saturated | 26:0,  30:0,  32:0,  34:0,  36:0,  38:0,  40:0,  42:0 | 24:0,  26:0,  30:0,  32:0,  34:0 | 24:0,  26:0,  30:0,  32:0,  34:0,  36:0,  38:0,  42:0 | 33:0,  34:0,  35:0,  36:0 | 15:0,  16:0,  18:0,  20:0,  22:0 | 12:0,  14:0,  15:0,  16:0,  18:0,  20:0,  22:0,  24:0,  26:0 | 8:0,  16:0,  18:0,  22:0,  23:0,  24:0 | 14:0,  15:0,  16:0,  18:0,  19:0,  20:0,  24:0, |
| Mono-unsaturated | 30:1,  32:1,  34:1,  36:1,  38:1,  40:1,  44:1 | 30:1,  32:1,  34:1,  36:1,  38:1,  40:1 | 32:1,  34:1,  36:1,  38:1,  40:1,  42:1 | 32:1,  33:1,  34:1,  35:1,  36:1 | 16:1,  18:1 | 16:1,  18:1,  20:1,  22:1,  23:1,  24:1,  26:1 | 18:1,  22:1,  24:1 | 14:1,  15:1,  16:1,  17:1,  18:1,  19:1,  20:1,  22:1,  24:1 |
| Poly-unsaturated | 32:3/2,  34:4/3/2  36:5/4/3/2 | 30:3/2,  32:3/2,  34:4/3/2,  36:6/5/4/3/2,  38:7/6/5/4/3/2,  40:6/5/4,  42:7/6/5 | 30:3,  32:2,  34:2,  36:4/3/2,  38:6/5/4/3/2,  40:7/6/5/4/3/2,  42:7/6/5/4/3/2 | 32:2,  34:3/2,  35:2,  36:4/3/2,  38:6/5/4/3/2,  40:7/6/5/4/3 | 18:3/2,  20:5/4/3,  22:6/5/4 | 18:2,  20:3,  22:4/3/2,  24:3/2,  26:4/3/2,  27:3,  28:5/4/3/2 |  | 16:3/2,  18:4/3/2,  19:2,  20:5/4/3/2,  22:6/5/4/3/2,  24:7/6/5/4/3/2 |
