# Supplementary material for: NMR Spectroscopy of Macrophages Loaded with Native, Oxidized or Enzymatically Degraded Lipoproteins
Source: PLoS One. 2013 Feb 15;8(2):e56360. doi: 10.1371/journal.pone.0056360 (PMC3574142; doi:10.1371/journal.pone.0056360)
Supplement: Table S2 — Resonance assignment used for deconvolution of NMR spectra of macrophage suspensions. Lineshape abbreviations: singlet (s), doublet (d), multiplet (m), broad peak treated as singlet (b), multiplet treated as broad singlet (m/b). (DOCX) [file pone.0056360.s003.docx]

## Supporting Table S2

**Resonance assignment used for deconvolution of NMR spectra of macrophage suspensions**. Lineshape abbreviations: singlet (s), doublet (d), multiplet (m), broad peak treated as singlet (b), multiplet treated as broad singlet (m / b).

| **Chemical shift [ppm]** | **Compound name** | **Line shape** | **Abbreviation** | **Remarks** |
| --- | --- | --- | --- | --- |
| 0.89 | Mobile lipid methyl groups | b | -CH_3_ (ML1) | Terminal group of fatty acid chains in mobile lipids |
| 0.87 & 0.93 | Mobile protein branched chain amino acid residues | b & b | M1 or MP1 |  |
| 0.90 – 1.05 | Branched-chain amino acids | m | BCAA | Valine, leucine and isoleucine |
| 1.21 | Mobile protein threonine residues | b | M2 or MP2 | Minor contributions of mobile protein isoleucine residues |
| 1.28 | Mobile lipid methylene groups | b | -CH_2_-C**H**_2_-CH_2_- (ML2) | Methylene groups neighbored by methylene groups within fatty acid chains |
| 1.32 | Lactate | d | lac |  |
| 1.39 | Mobile protein alanine residues | b | M3 or MP3 | Minor contributions of mobile protein lysine and isoleucine residues |
| 1.47 | Alanine | d | ala |  |
| 1.58 | Mobile lipid methylene groups | b | -CO-CH_2_-C**H**_2_- (ML3) | Second methylene group of fatty acid chains (near carboxyl group) |
| 1.68 | Mobile protein leucine, lysine and arginine residues | b | M4 or MP4 |  |
| 1.76 | Dimethyl-silapentane-sulfonate | m / b | DSS | Added chemical shift standard |
| 1.91 | Acetate | s | ace | Impurity |
| 2.03 | Mobile lipid methylene groups | b | =CH-C**H_2_-**CH_2_- (ML4) | Methylene groups next to one (!) double bonded methine pair within unsaturated fatty acid chains |
| 1.93 | Mobile protein glutamine, glutamate and proline residues | b | M5 or MP5 |  |
| 1.95 – 2.40 | Glutamine, glutamate, proline, glutathione | m / b | glx |  |
| 2.24 | Mobile lipid methylene groups | b | -CO-C**H**_2_- (ML5) | First methylene group of fatty acid chains (next to carboxyl group) |
| 2.06 & 2.15 | Mobile protein glutamine, glutamate and proline residues | b & b | M6 or MP6 |  |
| 2.54 | Glutathione | m / b | GSH |  |
| 2.79 | Mobile lipid methylene groups | b | =CH-C**H**_2_-CH= (ML6) | *Bis*-allylic methylene groups (surrounded by double bonded methine pairs) within poly-unsaturated fatty acid chains |
